# *catena*-Poly[[tetra­kis(μ_2_-acetato-κ^2^ *O*:*O*′)dicopper(II)(*Cu*—*Cu*)]-μ_2_-acetato-κ^2^ *O*:*O*′-[bis­[μ_2_-3-(dimethyl­amino)propan-1-olato]-κ^2^ *N*,*O*:*O*;κ^2^ *O*:*N*,*O*-bis­[(tetra­hydro­furan-κ*O*)copper(II)]]-μ_2_-acetato-κ^2^ *O*:*O*′]

**DOI:** 10.1107/S1600536808024148

**Published:** 2008-08-09

**Authors:** Muhammad Shahid, Muhammad Mazhar, Madeleine Helliwell, Javeed Akhtar, Kibriya Ahmad

**Affiliations:** a Department of Chemistry, Quaid-i-Azam University, Islamabad 45320, Pakistan; b The School of Chemistry, The University of Manchester, Oxford Road, Manchester M13 9PL, England

## Abstract

The title complex, [Cu_4_(C_5_H_12_NO)_2_(C_2_H_3_O_2_)_6_(C_4_H_8_O)_2_]_*n*_, consists of dinuclear [Cu_2_(C_5_H_12_NO)_2_(THF)_2_] (THF is tetra­hydro­furan) and [Cu_2_(CH_3_COO)_4_] units linked through acetate ions, generating parallel one-dimensional polymeric chains propagating in the [1
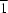
0] direction. In the first dinuclear unit, Cu^II^ ions related by inversion symmetry are bridged by two 3-(dimethyl­amino)propan-1-olate ligands. Likewise, a pair of inversion-related Cu^II^ ions are bridged by four acetate groups. The crystallographically independent Cu centers are linked to one another by a single bridging acetate group, generating an infinite chain. The distorted square-pyramidal coordination of the first metal center is completed with an apical THF mol­ecule, with a long Cu—O bond length of 2.476 (5) Å. The geometry around the other metal atom is close to octa­hedral, and the Cu⋯Cu separation in this unit is 2.652 (1) Å. The distance between the metal centers in the first dinuclear unit is considerably longer [3.068 (1) Å], suggesting little or no bonding inter­action. The Cu⋯Cu separation between two acetate-bridged independent Cu centers is 4.860 (2) Å. The THF mol­ecule has methyl­ene groups disordered over two positions, with occupancies of 0.608 (13) and 0.392 (13).

## Related literature

For related literature, see: Catania *et al.* (1990 ▶); El Fallah *et al.* (2004 ▶); Li *et al.* (1994 ▶); Mazhar *et al.* (2006 ▶); Tahir *et al.* (2007 ▶); Torres *et al.* (1996 ▶); Wang *et al.* (1993 ▶); Zhang *et al.* (2004 ▶).
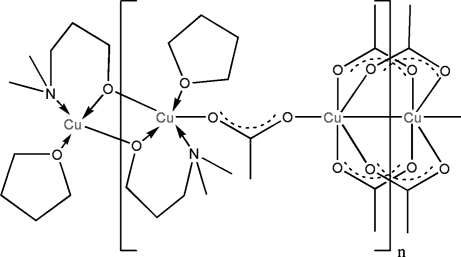


## Experimental

### 

#### Crystal data

[Cu_4_(C_5_H_12_NO)_2_(C_2_H_3_O_2_)_6_(C_4_H_8_O)_2_]
*M* *_r_* = 956.94
Monoclinic, 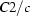

*a* = 25.686 (5) Å
*b* = 8.972 (5) Å
*c* = 18.021 (5) Å
β = 105.782 (5)°
*V* = 3996 (3) Å^3^
*Z* = 4
Mo *K*α radiation
μ = 2.17 mm^−1^
*T* = 100 (2) K
0.23 × 0.10 × 0.04 mm

#### Data collection

Bruker SMART CCD area-detector diffractometer
Absorption correction: none
15315 measured reflections
4093 independent reflections
3300 reflections with *I* > 2σ(*I*)
*R*_int_ = 0.093

#### Refinement

*R*[*F* ^2^ > 2σ(*F* ^2^)] = 0.075
*wR*(*F* ^2^) = 0.156
*S* = 1.22
4093 reflections
268 parameters
167 restraints
H-atom parameters constrained
Δρ_max_ = 1.17 e Å^−3^
Δρ_min_ = −0.89 e Å^−3^

### 

Data collection: *SMART* (Bruker, 2001 ▶); cell refinement: *SAINT* (Bruker, 2002 ▶); data reduction: *SAINT*; program(s) used to solve structure: *SHELXS97* (Sheldrick, 2008 ▶); program(s) used to refine structure: *SHELXL97* (Sheldrick, 2008 ▶); molecular graphics: *SHELXTL* (Sheldrick, 2008 ▶); software used to prepare material for publication: *SHELXTL*.

## Supplementary Material

Crystal structure: contains datablocks I, global. DOI: 10.1107/S1600536808024148/bh2182sup1.cif

Structure factors: contains datablocks I. DOI: 10.1107/S1600536808024148/bh2182Isup2.hkl

Additional supplementary materials:  crystallographic information; 3D view; checkCIF report

## Figures and Tables

**Fig. 1.**
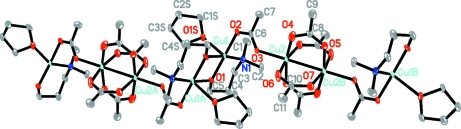
Molecular structure of (I) showing atom-labeling scheme and displacement ellipsoids at the 50% probability level. H atoms and disordered atoms have been removed for clarity. Symmetry codes: A 1/2-x, 1/2-y, 1-z; B 1-x, -y, 1-z.

## supplementary crystallographic information

### Comment

For the last two decades, there has been a considerable interest in the investigation of Cu^II^ complexes using carboxylate and aminoalcohol as ligands (Wang *et al.*, 1993; El Fallah *et al.*, 2004; Tahir *et al.*, 2007; Mazhar *et al.*, 2006) due to their potential use as CVD precursors for the deposition of copper and copper(II) oxide with diverse applications. For example, copper oxide is a component of high *T_c_* superconductors (Catania *et al.*, 1990). Being an excellent electrical conductor and good resistor for electromigration, metallic copper may replace aluminum alloys for multi-level metallization applications in Ultra Large Scale Integration (ULSI) technology (Torres *et al.*, 1996; Li *et al.*, 1994).

Herein, we report, the structure of a new polymeric Cu^II^ complex, (I), in which the molecules are linked into parallel, one dimensional polymeric chains in the [1 -1 0] direction, through acetate and 3-dimethylaminopropan-1-olate (dmap) bridging ligands, with tetrahydrofuran (THF) as an ancillary ligand.

In the title compound (Fig. 1), the environment of Cu1 is distorted square pyramidal, with the ligation set [CuO_4_N] consisting of O1, O1^i^ [symmetry code: (i) 1/2-*x*, 1/2-*y*, 1-*z*] of two bridging-chelating dmap ligands, O2 of the bridging acetate group, O1S of the THF molecule and N1 of one bridging-chelating dmap ligand. The deviation from perfect square pyramidal geometry around Cu1 is evident from the O1—Cu1—O1^i^ bite angle of 75.99 (18)° (less than 90°) and O1—Cu1—O2 angle, 167.93 (17)° [less than 180°]. Additionally, the Cu—O bond length of Cu1 to the apical O atom, O1S, is 2.476 (5) Å, longer than all other Cu—O bond lengths around Cu1, which are consistent with the sum of the ionic radii, 1.92 Å. The overall geometry around Cu2 is close to octahedral. The equatorial plane is formed by O4, O6, O5^ii^ and O7^ii^ of four bridging acetate groups connecting Cu2 and Cu2^ii^ [symmetry code: (ii) 1-*x*, -*y*, 1-*z*] atoms of two monomers in the polymeric structure, while O atom O3 of the bridging acetate links Cu2 in the axial position in the octahedron. The *trans* angles in the equatorial plane deviate slightly from ideal value of 180°, and the Cu2—O3 bond length of 2.141 (4) Å is slightly longer than the normal value (1.92 Å), indicating slightly distorted coordination geometry around the Cu2 atom in the complex. All the Cu2—O bond distances in the equatorial plane are in agreement with the bond lengths found in similar complexes (Zhang *et al.*, 2004). The inversion related Cu2 atoms are linked by Cu—Cu bonds of 2.652 (1) Å, completing the octahedral coordination of Cu2. The distance between the inversion related Cu1 atoms is considerably longer, 3.068 (1) Å, suggesting little or no bonding interaction, and the Cu1···Cu2 separation is 4.860 (2) Å.

### Experimental

3-Dimethylamino-1-propanol (0.15 g, 1.25 mmol) was added to a stirred suspension of Cu(CH_3_COO)_2_.H_2_O (0.50 g, 2.50 mmol) in 25 ml of THF. After two hours of stirring, the mixture was vacuum evaporated to dryness and the resulting solid was redissolved in THF to give greenish blue block-shaped crystals, at room temperature, after 10 days.

### Refinement

Atoms C1S, C2S and C3S of the THF molecule are disordered over two sites with occupancies constrained to sum to unity, and the highest occupancy fraction refining to a value of 0.608 (13). Distance restraints were applied to the O—C and C—C bond lengths of the disordered THF using the SADI command and restraints were applied to the atomic displacement parameters of this molecule. H atoms were included in calculated positions using the riding model with C—H distances ranging from 0.96 to 0.97 Å and *U*_eq _values 1.2 to 1.5 times those of the parent atoms; the methyl H atoms were calculated so as to maximize the sum of the electron density at the three calculated positions.

### Crystal data

**Table:**

[Cu_4_(C_5_H_12_NO)_2_(C_2_H_3_O_2_)_6_(C_4_H_8_O)_2_]	*F*_000_ = 1984
*M**_r_* = 956.94	*D*_x_ = 1.590 Mg m^−^^3^
Monoclinic, *C*2/*c*	Mo *K*α radiation λ = 0.71069 Å
Hall symbol: -C 2yc	Cell parameters from 2913 reflections
*a* = 25.686 (5) Å	θ = 2.4–26.4º
*b* = 8.972 (5) Å	µ = 2.17 mm^−^^1^
*c* = 18.021 (5) Å	*T* = 100 (2) K
β = 105.782 (5)º	Plate, blue
*V* = 3996 (3) Å^3^	0.23 × 0.10 × 0.04 mm
*Z* = 4	

### Data collection

**Table:**

Bruker SMART CCD area-detector diffractometer	3300 reflections with *I* > 2σ(*I*)
Radiation source: fine-focus sealed tube	*R*_int_ = 0.093
Monochromator: graphite	θ_max_ = 26.4º
*T* = 100(2) K	θ_min_ = 2.4º
φ and ω scans	*h* = −32→32
Absorption correction: none	*k* = −11→11
15315 measured reflections	*l* = −22→21
4093 independent reflections	

### Refinement

**Table:**

Refinement on *F*^2^	Secondary atom site location: difference Fourier map
Least-squares matrix: full	Hydrogen site location: inferred from neighbouring sites
*R*[*F*^2^ > 2σ(*F*^2^)] = 0.075	H-atom parameters constrained
*wR*(*F*^2^) = 0.156	*w* = 1/[σ^2^(*F*_o_^2^) + (0.0593*P*)^2^ + 6.3428*P*] where *P* = (*F*_o_^2^ + 2*F*_c_^2^)/3
*S* = 1.22	(Δ/σ)_max_ < 0.001
4093 reflections	Δρ_max_ = 1.17 e Å^−^^3^
268 parameters	Δρ_min_ = −0.89 e Å^−^^3^
167 restraints	Extinction correction: none
Primary atom site location: structure-invariant direct methods	

### Fractional atomic coordinates and isotropic or equivalent isotropic displacement parameters (Å^2^)

**Table:**

	*x*	*y*	*z*	*U*_iso_*/*U*_eq_	Occ. (<1)
Cu1	0.30186 (3)	0.30456 (8)	0.55814 (4)	0.0138 (2)	
Cu2	0.45657 (3)	0.02909 (8)	0.52386 (4)	0.0172 (2)	
O1	0.25020 (14)	0.3612 (4)	0.4632 (2)	0.0151 (8)	
O2	0.35126 (14)	0.2046 (5)	0.6459 (2)	0.0176 (9)	
O3	0.38789 (15)	0.0922 (5)	0.5624 (2)	0.0185 (9)	
O4	0.48175 (19)	−0.1399 (6)	0.5945 (3)	0.0396 (13)	
O5	0.55563 (17)	−0.1852 (5)	0.5562 (2)	0.0256 (10)	
O6	0.42008 (17)	−0.1147 (6)	0.4445 (3)	0.0351 (12)	
O7	0.49313 (17)	−0.1635 (5)	0.4038 (3)	0.0291 (11)	
N1	0.35282 (19)	0.4863 (5)	0.5650 (3)	0.0183 (11)	
C1	0.3780 (3)	0.5304 (8)	0.6455 (4)	0.0299 (15)	
H1A	0.4026	0.6114	0.6466	0.045*	
H1B	0.3973	0.4472	0.6734	0.045*	
H1C	0.3504	0.5614	0.6689	0.045*	
C2	0.3966 (2)	0.4443 (7)	0.5296 (4)	0.0238 (14)	
H2A	0.4181	0.5305	0.5265	0.036*	
H2B	0.3810	0.4057	0.4787	0.036*	
H2C	0.4191	0.3694	0.5606	0.036*	
C3	0.3233 (2)	0.6201 (7)	0.5256 (4)	0.0236 (14)	
H3A	0.3493	0.6994	0.5275	0.028*	
H3B	0.2985	0.6534	0.5542	0.028*	
C4	0.2913 (2)	0.5957 (7)	0.4414 (4)	0.0256 (15)	
H4A	0.2821	0.6919	0.4168	0.031*	
H4B	0.3141	0.5439	0.4148	0.031*	
C5	0.2399 (2)	0.5068 (7)	0.4328 (4)	0.0235 (14)	
H5A	0.2169	0.5586	0.4591	0.028*	
H5B	0.2206	0.5005	0.3786	0.028*	
C6	0.3837 (2)	0.1105 (7)	0.6289 (3)	0.0180 (13)	
C7	0.4177 (3)	0.0192 (8)	0.6959 (4)	0.0328 (17)	
H7A	0.4166	−0.0839	0.6813	0.049*	
H7B	0.4035	0.0299	0.7397	0.049*	
H7C	0.4544	0.0540	0.7090	0.049*	
C8	0.5252 (2)	−0.2083 (7)	0.5982 (4)	0.0209 (13)	
C9	0.5405 (3)	−0.3270 (7)	0.6589 (4)	0.0319 (16)	
H9A	0.5155	−0.4085	0.6456	0.048*	
H9B	0.5394	−0.2866	0.7078	0.048*	
H9C	0.5764	−0.3620	0.6623	0.048*	
C10	0.4439 (2)	−0.1799 (7)	0.4023 (4)	0.0211 (13)	
C11	0.4112 (3)	−0.2912 (7)	0.3460 (4)	0.0311 (16)	
H11A	0.3992	−0.3694	0.3738	0.047*	
H11B	0.4332	−0.3328	0.3158	0.047*	
H11C	0.3804	−0.2424	0.3126	0.047*	
O1S	0.25794 (16)	0.4312 (6)	0.6472 (2)	0.0302 (10)	
C1S	0.2630 (5)	0.3409 (16)	0.7175 (6)	0.031 (2)	0.608 (13)
H1S1	0.2811	0.3983	0.7628	0.037*	0.608 (13)
H1S2	0.2843	0.2524	0.7159	0.037*	0.608 (13)
C2S	0.2070 (5)	0.2968 (15)	0.7220 (8)	0.035 (2)	0.608 (13)
H2S1	0.1953	0.2038	0.6952	0.042*	0.608 (13)
H2S2	0.2054	0.2885	0.7750	0.042*	0.608 (13)
C3S	0.1749 (4)	0.4257 (14)	0.6819 (6)	0.0286 (18)	0.608 (13)
H3S1	0.1781	0.5108	0.7161	0.034*	0.608 (13)
H3S2	0.1370	0.3998	0.6620	0.034*	0.608 (13)
C4S	0.2002 (2)	0.4563 (8)	0.6182 (4)	0.0272 (12)	0.608 (13)
H4S1	0.1851	0.3906	0.5749	0.033*	0.608 (13)
H4S2	0.1933	0.5585	0.6007	0.033*	0.608 (13)
C1T	0.2742 (8)	0.392 (3)	0.7265 (8)	0.034 (3)	0.392 (13)
H1T1	0.2724	0.4753	0.7599	0.041*	0.392 (13)
H1T2	0.3099	0.3474	0.7417	0.041*	0.392 (13)
C2T	0.2304 (7)	0.281 (2)	0.7224 (11)	0.037 (3)	0.392 (13)
H2T1	0.2230	0.2718	0.7722	0.044*	0.392 (13)
H2T2	0.2410	0.1843	0.7075	0.044*	0.392 (13)
C3T	0.1812 (6)	0.337 (2)	0.6632 (9)	0.030 (2)	0.392 (13)
H3T1	0.1552	0.3768	0.6881	0.036*	0.392 (13)
H3T2	0.1642	0.2560	0.6293	0.036*	0.392 (13)
C4T	0.2002 (2)	0.4563 (8)	0.6182 (4)	0.0272 (12)	0.392 (13)
H4T1	0.1874	0.4401	0.5630	0.033*	0.392 (13)
H4T2	0.1899	0.5551	0.6307	0.033*	0.392 (13)

### Atomic displacement parameters (Å^2^)

**Table:**

	*U*^11^	*U*^22^	*U*^33^	*U*^12^	*U*^13^	*U*^23^
Cu1	0.0061 (3)	0.0192 (4)	0.0153 (4)	0.0016 (3)	0.0012 (3)	−0.0012 (3)
Cu2	0.0079 (3)	0.0222 (4)	0.0218 (4)	0.0048 (3)	0.0045 (3)	0.0020 (3)
O1	0.0100 (18)	0.015 (2)	0.017 (2)	0.0006 (16)	−0.0010 (16)	0.0048 (17)
O2	0.0077 (19)	0.026 (2)	0.017 (2)	0.0038 (16)	0.0001 (16)	−0.0036 (18)
O3	0.0070 (18)	0.032 (3)	0.016 (2)	0.0028 (17)	0.0023 (16)	0.0001 (18)
O4	0.028 (3)	0.043 (3)	0.056 (3)	0.021 (2)	0.026 (3)	0.028 (3)
O5	0.018 (2)	0.035 (3)	0.026 (2)	0.0129 (19)	0.0088 (19)	0.008 (2)
O6	0.013 (2)	0.044 (3)	0.050 (3)	−0.006 (2)	0.010 (2)	−0.022 (3)
O7	0.017 (2)	0.045 (3)	0.025 (2)	−0.003 (2)	0.0047 (19)	−0.010 (2)
N1	0.012 (2)	0.021 (3)	0.024 (3)	−0.002 (2)	0.007 (2)	−0.003 (2)
C1	0.025 (3)	0.035 (4)	0.024 (4)	−0.004 (3)	−0.004 (3)	−0.007 (3)
C2	0.013 (3)	0.024 (4)	0.037 (4)	0.001 (2)	0.011 (3)	−0.002 (3)
C3	0.014 (3)	0.020 (3)	0.035 (4)	0.001 (2)	0.002 (3)	−0.001 (3)
C4	0.016 (3)	0.019 (3)	0.039 (4)	−0.003 (2)	0.002 (3)	0.009 (3)
C5	0.013 (3)	0.027 (4)	0.028 (3)	0.000 (2)	0.001 (3)	0.002 (3)
C6	0.007 (3)	0.022 (3)	0.023 (3)	−0.003 (2)	0.000 (2)	0.001 (3)
C7	0.027 (4)	0.044 (4)	0.027 (4)	0.021 (3)	0.007 (3)	0.010 (3)
C8	0.018 (3)	0.022 (3)	0.020 (3)	−0.001 (2)	0.001 (2)	0.000 (3)
C9	0.027 (4)	0.028 (4)	0.042 (4)	0.010 (3)	0.012 (3)	0.014 (3)
C10	0.018 (3)	0.018 (3)	0.025 (3)	0.003 (2)	0.003 (3)	0.007 (3)
C11	0.030 (4)	0.026 (4)	0.033 (4)	−0.004 (3)	0.001 (3)	−0.002 (3)
O1S	0.0107 (18)	0.057 (3)	0.024 (2)	0.0075 (19)	0.0063 (17)	−0.003 (2)
C1S	0.014 (4)	0.055 (5)	0.021 (4)	0.011 (4)	0.000 (3)	−0.002 (4)
C2S	0.020 (4)	0.049 (5)	0.034 (4)	0.005 (4)	0.006 (4)	0.010 (3)
C3S	0.012 (3)	0.042 (4)	0.032 (4)	0.002 (3)	0.007 (3)	0.002 (4)
C4S	0.011 (2)	0.041 (3)	0.029 (2)	0.008 (2)	0.0047 (19)	0.004 (2)
C1T	0.015 (4)	0.060 (6)	0.023 (4)	0.008 (4)	−0.002 (4)	−0.003 (5)
C2T	0.025 (5)	0.052 (5)	0.030 (5)	0.008 (5)	0.001 (4)	0.008 (4)
C3T	0.015 (4)	0.045 (5)	0.030 (4)	0.004 (4)	0.006 (4)	0.003 (4)
C4T	0.011 (2)	0.041 (3)	0.029 (2)	0.008 (2)	0.0047 (19)	0.004 (2)

### Geometric parameters (Å, °)

**Table:**

Cu1—O1	1.926 (4)	C5—H5B	0.9700
Cu1—O2	1.956 (4)	C6—C7	1.522 (8)
Cu1—O1^i^	1.967 (4)	C7—H7A	0.9600
Cu1—N1	2.074 (5)	C7—H7B	0.9600
Cu2—O6	1.965 (5)	C7—H7C	0.9600
Cu2—O4	1.972 (5)	C8—C9	1.501 (8)
Cu2—O5^ii^	1.974 (4)	C9—H9A	0.9600
Cu2—O7^ii^	1.977 (4)	C9—H9B	0.9600
Cu2—O3	2.141 (4)	C9—H9C	0.9600
Cu2—Cu2^ii^	2.6520 (14)	C10—C11	1.506 (9)
O1—C5	1.414 (7)	C11—H11A	0.9600
O1—Cu1^i^	1.967 (4)	C11—H11B	0.9600
O2—C6	1.280 (7)	C11—H11C	0.9600
O3—C6	1.243 (7)	O1S—C1T	1.420 (13)
O4—C8	1.260 (7)	O1S—C4S	1.449 (6)
O5—C8	1.244 (7)	O1S—C1S	1.480 (10)
O5—Cu2^ii^	1.974 (4)	C1S—C2S	1.514 (12)
O6—C10	1.244 (7)	C1S—H1S1	0.9700
O7—C10	1.265 (7)	C1S—H1S2	0.9700
O7—Cu2^ii^	1.977 (4)	C2S—C3S	1.489 (11)
N1—C1	1.473 (8)	C2S—H2S1	0.9700
N1—C2	1.485 (7)	C2S—H2S2	0.9700
N1—C3	1.492 (8)	C3S—C4S	1.493 (10)
C1—H1A	0.9600	C3S—H3S1	0.9700
C1—H1B	0.9600	C3S—H3S2	0.9700
C1—H1C	0.9600	C4S—H4S1	0.9700
C2—H2A	0.9600	C4S—H4S2	0.9700
C2—H2B	0.9600	C1T—C2T	1.487 (14)
C2—H2C	0.9600	C1T—H1T1	0.9700
C3—C4	1.532 (9)	C1T—H1T2	0.9700
C3—H3A	0.9700	C2T—C3T	1.499 (14)
C3—H3B	0.9700	C2T—H2T1	0.9700
C4—C5	1.514 (8)	C2T—H2T2	0.9700
C4—H4A	0.9700	C3T—H3T1	0.9700
C4—H4B	0.9700	C3T—H3T2	0.9700
C5—H5A	0.9700		
			
O1—Cu1—O2	167.93 (17)	O3—C6—C7	120.8 (5)
O1—Cu1—O1^i^	75.99 (18)	O2—C6—C7	115.8 (5)
O2—Cu1—O1^i^	93.93 (16)	C6—C7—H7A	109.5
O1—Cu1—N1	96.69 (18)	C6—C7—H7B	109.5
O2—Cu1—N1	93.02 (18)	H7A—C7—H7B	109.5
O1^i^—Cu1—N1	172.24 (18)	C6—C7—H7C	109.5
O6—Cu2—O4	88.5 (2)	H7A—C7—H7C	109.5
O6—Cu2—O5^ii^	89.1 (2)	H7B—C7—H7C	109.5
O4—Cu2—O5^ii^	167.51 (18)	O5—C8—O4	125.5 (6)
O6—Cu2—O7^ii^	167.87 (18)	O5—C8—C9	118.6 (5)
O4—Cu2—O7^ii^	90.1 (2)	O4—C8—C9	115.9 (5)
O5^ii^—Cu2—O7^ii^	89.7 (2)	C8—C9—H9A	109.5
O6—Cu2—O3	97.94 (17)	C8—C9—H9B	109.5
O4—Cu2—O3	98.52 (17)	H9A—C9—H9B	109.5
O5^ii^—Cu2—O3	93.94 (16)	C8—C9—H9C	109.5
O7^ii^—Cu2—O3	94.19 (17)	H9A—C9—H9C	109.5
O6—Cu2—Cu2^ii^	84.91 (13)	H9B—C9—H9C	109.5
O4—Cu2—Cu2^ii^	84.33 (13)	O6—C10—O7	125.9 (6)
O5^ii^—Cu2—Cu2^ii^	83.26 (12)	O6—C10—C11	116.5 (5)
O7^ii^—Cu2—Cu2^ii^	82.96 (13)	O7—C10—C11	117.6 (6)
O3—Cu2—Cu2^ii^	176.00 (12)	C10—C11—H11A	109.5
C5—O1—Cu1	126.8 (4)	C10—C11—H11B	109.5
C5—O1—Cu1^i^	125.3 (3)	H11A—C11—H11B	109.5
Cu1—O1—Cu1^i^	104.01 (18)	C10—C11—H11C	109.5
C6—O2—Cu1	115.5 (4)	H11A—C11—H11C	109.5
C6—O3—Cu2	130.0 (4)	H11B—C11—H11C	109.5
C8—O4—Cu2	122.6 (4)	C1T—O1S—C4S	113.2 (10)
C8—O5—Cu2^ii^	124.2 (4)	C4S—O1S—C1S	103.7 (6)
C10—O6—Cu2	122.5 (4)	O1S—C1S—C2S	109.0 (8)
C10—O7—Cu2^ii^	123.7 (4)	O1S—C1S—H1S1	109.9
C1—N1—C2	108.1 (5)	C2S—C1S—H1S1	109.9
C1—N1—C3	106.4 (5)	O1S—C1S—H1S2	109.9
C2—N1—C3	110.3 (5)	C2S—C1S—H1S2	109.9
C1—N1—Cu1	111.7 (4)	H1S1—C1S—H1S2	108.3
C2—N1—Cu1	108.3 (4)	C3S—C2S—C1S	100.4 (9)
C3—N1—Cu1	111.9 (3)	C3S—C2S—H2S1	111.7
N1—C1—H1A	109.5	C1S—C2S—H2S1	111.7
N1—C1—H1B	109.5	C3S—C2S—H2S2	111.7
H1A—C1—H1B	109.5	C1S—C2S—H2S2	111.7
N1—C1—H1C	109.5	H2S1—C2S—H2S2	109.5
H1A—C1—H1C	109.5	C2S—C3S—C4S	102.4 (8)
H1B—C1—H1C	109.5	C2S—C3S—H3S1	111.3
N1—C2—H2A	109.5	C4S—C3S—H3S1	111.3
N1—C2—H2B	109.5	C2S—C3S—H3S2	111.3
H2A—C2—H2B	109.5	C4S—C3S—H3S2	111.3
N1—C2—H2C	109.5	H3S1—C3S—H3S2	109.2
H2A—C2—H2C	109.5	O1S—C4S—C3S	108.2 (6)
H2B—C2—H2C	109.5	O1S—C4S—H4S1	110.1
N1—C3—C4	115.0 (5)	C3S—C4S—H4S1	110.1
N1—C3—H3A	108.5	O1S—C4S—H4S2	110.1
C4—C3—H3A	108.5	C3S—C4S—H4S2	110.1
N1—C3—H3B	108.5	H4S1—C4S—H4S2	108.4
C4—C3—H3B	108.5	O1S—C1T—C2T	96.0 (13)
H3A—C3—H3B	107.5	O1S—C1T—H1T1	112.5
C5—C4—C3	113.2 (5)	C2T—C1T—H1T1	112.5
C5—C4—H4A	108.9	O1S—C1T—H1T2	112.5
C3—C4—H4A	108.9	C2T—C1T—H1T2	112.5
C5—C4—H4B	108.9	H1T1—C1T—H1T2	110.1
C3—C4—H4B	108.9	C1T—C2T—C3T	107.0 (14)
H4A—C4—H4B	107.7	C1T—C2T—H2T1	110.3
O1—C5—C4	112.4 (5)	C3T—C2T—H2T1	110.3
O1—C5—H5A	109.1	C1T—C2T—H2T2	110.3
C4—C5—H5A	109.1	C3T—C2T—H2T2	110.3
O1—C5—H5B	109.1	H2T1—C2T—H2T2	108.6
C4—C5—H5B	109.1	C2T—C3T—H3T1	110.4
H5A—C5—H5B	107.9	C2T—C3T—H3T2	110.4
O3—C6—O2	123.4 (5)	H3T1—C3T—H3T2	108.6
			
O2—Cu1—O1—C5	−167.5 (7)	C1—N1—C3—C4	−176.8 (5)
O1^i^—Cu1—O1—C5	158.4 (5)	C2—N1—C3—C4	66.2 (6)
N1—Cu1—O1—C5	−24.2 (5)	Cu1—N1—C3—C4	−54.5 (6)
O2—Cu1—O1—Cu1^i^	34.0 (8)	N1—C3—C4—C5	74.3 (7)
O1^i^—Cu1—O1—Cu1^i^	0.001 (1)	Cu1—O1—C5—C4	41.8 (7)
N1—Cu1—O1—Cu1^i^	177.36 (19)	Cu1^i^—O1—C5—C4	−164.1 (4)
O1—Cu1—O2—C6	49.2 (9)	C3—C4—C5—O1	−62.2 (7)
O1^i^—Cu1—O2—C6	82.2 (4)	Cu2—O3—C6—O2	148.6 (4)
N1—Cu1—O2—C6	−94.4 (4)	Cu2—O3—C6—C7	−31.7 (8)
O6—Cu2—O3—C6	136.6 (5)	Cu1—O2—C6—O3	6.6 (7)
O4—Cu2—O3—C6	46.9 (6)	Cu1—O2—C6—C7	−173.2 (4)
O5^ii^—Cu2—O3—C6	−133.8 (5)	Cu2^ii^—O5—C8—O4	2.4 (9)
O7^ii^—Cu2—O3—C6	−43.8 (5)	Cu2^ii^—O5—C8—C9	−177.7 (4)
O6—Cu2—O4—C8	88.2 (6)	Cu2—O4—C8—O5	−4.3 (10)
O5^ii^—Cu2—O4—C8	9.5 (14)	Cu2—O4—C8—C9	175.8 (5)
O7^ii^—Cu2—O4—C8	−79.7 (6)	Cu2—O6—C10—O7	−0.3 (9)
O3—Cu2—O4—C8	−174.0 (5)	Cu2—O6—C10—C11	177.8 (4)
Cu2^ii^—Cu2—O4—C8	3.2 (5)	Cu2^ii^—O7—C10—O6	0.6 (9)
O4—Cu2—O6—C10	−84.5 (5)	Cu2^ii^—O7—C10—C11	−177.5 (4)
O5^ii^—Cu2—O6—C10	83.3 (5)	C1T—O1S—C1S—C2S	127 (4)
O7^ii^—Cu2—O6—C10	−0.9 (14)	C4S—O1S—C1S—C2S	7.9 (13)
O3—Cu2—O6—C10	177.1 (5)	O1S—C1S—C2S—C3S	−29.8 (14)
Cu2^ii^—Cu2—O6—C10	0.0 (5)	C1S—C2S—C3S—C4S	38.9 (12)
O1—Cu1—N1—C1	146.9 (4)	C1T—O1S—C4S—C3S	−2.4 (15)
O2—Cu1—N1—C1	−40.3 (4)	C1S—O1S—C4S—C3S	17.8 (10)
O1—Cu1—N1—C2	−94.2 (4)	C2S—C3S—C4S—O1S	−37.0 (11)
O2—Cu1—N1—C2	78.7 (4)	C4S—O1S—C1T—C2T	45.5 (19)
O1—Cu1—N1—C3	27.7 (4)	C1S—O1S—C1T—C2T	−22 (3)
O2—Cu1—N1—C3	−159.5 (4)	O1S—C1T—C2T—C3T	−34 (2)

Symmetry codes: (i) −*x*+1/2, −*y*+1/2, −*z*+1; (ii) −*x*+1, −*y*, −*z*+1.
